# Comparative efficacy and safety of induction therapy in solid organ transplantation: a systematic review and network meta-analysis

**DOI:** 10.3389/fimmu.2025.1625710

**Published:** 2025-07-14

**Authors:** Junjie Sun, Chao Hu, Qingwen Liang, Yanqing Yu, Ning Wen, Jianhui Dong, Haibin Li, Xuyong Sun

**Affiliations:** Institute of Transplantation Medicine, The Second Affiliated Hospital of Guangxi Medical University, Guangxi Clinical Research Center for Organ Transplantation, Guangxi Key Laboratory of Organ Donation and Transplantation, Nanning, Guangxi, China

**Keywords:** organ transplantation, induction therapy, rejection, network meta-analysis, randomized controlled trials

## Abstract

**Objective:**

To comparatively evaluate the efficacy and safety of induction therapies in solid organ transplantation (SOT) using a Bayesian network meta-analysis (NMA).

**Methods:**

Randomized controlled trials (RCTs) assessing induction therapies were systematically identified across major databases (up to November 20, 2024). The screening, data extraction, and risk of bias (ROB) assessment were independently conducted by two reviewers through standardized tools. Bayesian NMA synthesized outcomes, including rejection, graft/overall survival, and infection rates.

**Results:**

Sixty-eight RCTs (9,626 patients) evaluating 12 therapies were included. Surface Under the Cumulative Ranking Area (SUCRA) probabilities identified alemtuzumab as the most effective agent for reducing rejection rates (93.9%), followed by antilymphocyte globulin (ALG, 87.0%) and belimumab (77.0%). For graft survival, OKT3 ranked highest (87.9%), with subsequent superiority for ALG (83.5%) and alemtuzumab (75.6%). Basiliximab demonstrated the highest overall survival benefit (88.0%), outperforming rabbit antithymocyte globulin (rATG, 82.1%) and inolimomab (70.3%). Belimumab showed the greatest infection risk reduction (94.4%), surpassing alemtuzumab (80.0%) and basiliximab (74.5%).

**Conclusion:**

Alemtuzumab emerged as the optimal therapy for minimizing rejection, while OKT3 and basiliximab were superior for graft and overall survival, respectively. Belimumab exhibited the strongest potential for reducing incidence of infection. These findings highlight therapy-specific advantages for optimizing SOT outcomes.

**Systematic review registration:**

https://www.crd.york.ac.uk/PROSPERO/myprospero, identifier CRD42025634120.

## 1 Introduction

Solid organ transplantation (SOT) has emerged as the optimal therapy for patients with end-stage organ disease, based on both clinical and economic perspectives (1). However, a significant challenge in this field is the persistent shortage of organs (2). Mortality rates for individuals remaining on the transplant waiting list continue to be significantly elevated (3). Consequently, enhancing graft survival remains a pressing issue requiring resolution. Acute rejection following organ transplantation is the primary determinant of short-term graft survival (4). Furthermore, prolonged administration of immunosuppressive medications can progressively impair graft function, ultimately leading to graft failure (5). Additionally, chronic antibody-mediated rejection driven by donor-specific antibodies (DSA) poses a major obstacle to the long-term survival of transplanted organs (1, 6). Nonetheless, antibody induction therapy has partially mitigated this issue. Numerous prospective RCTs have confirmed that antibody induction significantly decreases acute rejection episodes (4) and markedly enhances short-term graft survival (7). This improvement can be attributed to the widespread adoption of preoperative induction protocols, which facilitate delayed initiation of postoperative immunosuppressive regimens and allow for lower doses of maintenance immunosuppressants, thus reducing the harmful impact of immunosuppressive drugs on the graft (8). The landscape of induction therapy has evolved substantially over the past several decades. Initially, many transplantation centers explored either no induction or therapies involving cyclophosphamide or anti-CD3 monoclonal antibodies (OKT3) (9); however, these approaches have gradually been phased out because of their considerable side effects. Despite the potential promise of targeting B cell activation and acknowledging the role of DSA in SOT (10), current immunosuppressive research continues to focus primarily on T cells (11, 12). Agents such as antithymocyte globulin (ATG), anti-CD52, and anti-CD25 have gained widespread acceptance. However, the efficacy and safety of these induction protocols remain unclear. Therefore, establishing a widely accepted, safe, and effective pre-transplantation induction regimen is crucial for preventing graft damage and loss due to acute rejection.

Network meta-analysis (NMA) enables comparative assessments of therapeutic interventions through indirect treatment comparisons and quantitative synthesis, thereby facilitating the optimization of therapeutic strategies. In this investigation, we employ NMA methodology to systematically evaluate the efficacy of induction therapy in preventing acute rejection episodes among SOT recipients. The findings aim to provide evidence-based recommendations to inform clinical decision-making in this patient population.

## 2 Data and methods

This systematic review and NMA registers on PROSPERO. The work has been reported in line with PRISMA (Preferred Reporting Items for Systematic Reviews and Meta-Analyses) (13) and AMSTAR (Assessing the methodological quality of systematic reviews) Guidelines (14).

### 2.1 Literature screening

A comprehensive search was conducted in Cochrane Library, PubMed, Embase, and Web of Science (through November 20, 2024) using MeSH and free-text keywords such as “transplantation”, “rejection”, and “randomized controlled trials”. Full search syntax is available in **Supplementary Material 1**.

### 2.2 Eligibility criteria

#### 2.2.1 Type: randomized controlled trials (RCTs)

#### 2.2.2 Population: SOT patients receiving induction therapy

#### 2.2.3 Intervention

Induction therapies included basiliximab, alemtuzumab, daclizumab, horse anti-thymocyte globulin (hATG), rabbit anti-thymocyte globulin (rATG), odulimumab, inolimomab, OKT3, Lo-tact-1, rituximab, belimumab, and anti-lymphocyte globulin (ALG). The control group received standard care.

#### 2.2.4 Outcome

Primary outcome: Rejection rate.

Secondary outcomes: (1) Graft survival rate. (2) Overall survival rate. (3) Infection rate.

#### 2.2.5 Exclusion

(1) Non-English language publications (15). (2) Duplicate datasets. (3) Insufficient outcome reporting. (4) Unresolved data discrepancies after author contact. (5) Test or control groups receiving pharmacological agents for the treatment of rejection. (6) Cell transplantation and tissue transplantation.

### 2.3 Data collection

Two investigators independently screened titles/abstracts, reviewed full texts, and extracted data using standardized forms. Discrepancies were resolved via consensus or third-party adjudication. Corresponding authors were contacted for missing data. Extracted variables encompassed study metadata (authors, country, year), participant demographics, intervention protocols, and outcome metrics.

### 2.4 Quality assessment

Risk of bias (ROB) assessment for included RCTs followed Cochrane Collaboration guidelines (v5.1.0), evaluating seven domains: randomization, allocation concealment, blinding, incomplete data, selective reporting, and other biases. The category “Other biases” assesses whether significant factors exist in the specific study being evaluated, considering its design and methodological details, which might systematically distort its results and are not covered by the six standard domains. Examples include bias in crossover trials, contamination, bias due to early stopping, bias due to baseline imbalances, and recruitment bias in cluster-randomized trials. Two reviewers independently categorized each domain as high, unclear risk, or low. Disagreements were resolved through discussion.

### 2.5 Statistical methods

Bayesian NMA was performed using R 4.4.0 (GeMTC package) with model consistency verified via Deviance Information Criterion (DIC; **Supplementary Material 2**). Fixed-effects models generated mean differences (95% CIs). Results were visualized via forest plots, league tables, and ranking probability diagrams. Surface Under the Cumulative Ranking Area (SUCRA) scores (0–1 scale) ranked intervention efficacy. Stata 18 generated network plots and assessed publication bias via comparison-adjusted funnel plots.

## 3 Results

### 3.1 Study selection

**Figure 1:**
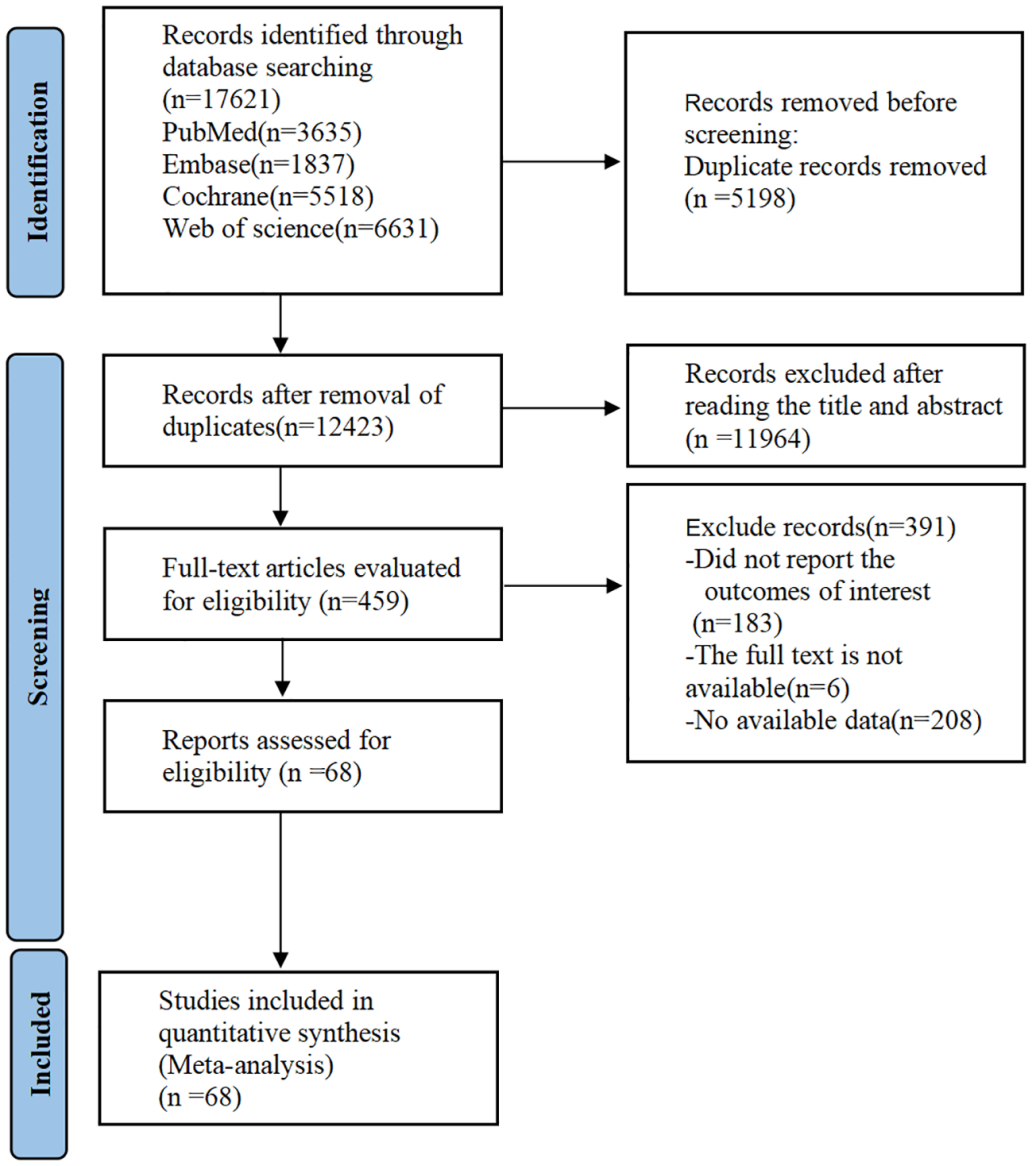
PRISMA flow diagram of the study process. PRISMA, Preferred Reporting Items for Meta-analysis.

Initial database screening identified 17,621 English-language records. After removing 5,198 duplicates, 11,964 studies were excluded through title/abstract screening. Full-text review excluded 391 additional articles, yielding 68 eligible RCTs for final inclusion (**Figure 1**).

### 3.2 Study characteristics

68 RCTs, comprising 9,626 patients, were included. Twelve different induction therapies were used: basiliximab, alemtuzumab, daclizumab, hATG, rATG, odulimomab, inolimomab, OKT3, Lo-tact-1, rituximab, belimumab, and ALG. The effects of each intervention were compared against the control group’s results in a pairwise fashion. The fundamental attributes of these studies are listed in **Table 1**.

**Table 1:** The basic characteristics of included studies.

Number	Study	Year	Country	Sample size	Gender(M/F)	Organ	Mean age	Intervention	Outcome
1	Stuart C. Sweet	2022	United States	Rituximab:15 Control(Placebo):12	13/14	Lung	Rituximab:12.5±4.55 Control:13.2±4.47	Rituximab:250 mg/m²,IV,on days 0,1,2; rATG:1.5 mg/kg/d,on days 0, 1,2;	F1:0/2 F4:11/11
2	Heshmatollah Shahbazian	2020	Iran	rATG:53 Control(Placebo):53	58/48	Kidney	rATG:38.74±12.33 Control:37.85±13.69	rATG:1.5 mg/kg/d,IV,on days 0, 1,2;	F1:8/11 F4:44/44 F5:36/33
3	Nassim Kamar	2020	France	ALG:32 Control(Basiliximab):27	22/37	Kidney	ALG:56.8±8.5 Control:53.0±13.6	ALG:1mg/kg,IV, on day 0;3 mg/kg on day 1,3,5; Basiliximab:20 mg,IV,on days 0,4;	F1:1/2 F3:29/24 F4:31/26 F5:23/18
4	Rita R. Alloway	2019	United States, France, Belgium	rATG:254 Contro(Basiliximab):254	322/186	Kidney	rATG:52.3 Control:51.6	rATG:1.5 mg/kg,IV,on days 0, 1,2;	F1:64/91 F3:226/228 F4:244/244 F5:86/69
5	Banham, Gemma D	2018	United Kingdom	Belimumab:13 Control(Placebo):12	14/11	Kidney	Belimumab:51± 14 Control:54.3± 11	Belimumab:10mg/kg,IV, on days 0, 14,28,and on weeks 8,12,16,20;	F1:1/3 F4:13/11 F5:1/5
6	M. W. F. van den Hoogen	2015	Netherlands	Rituximab:138 Control(Placebo):142	186/94	Kidney	Rituximab:50.8± 13.2 Control:49.8±12.3	Rituximab:375 mg/m²,IV,on days 0,1;	F1:23/30 F4:137/141 F5:88/88
7	R. Hellemans	2005	France, Belgium	rATG:106 Control(Daclizumab):104	106/104	Kidney	rATG:44.9±10.3 Control:47.3±9.0	rATG:1.25 mg/kg/day,IV,on days 0-7; Daclizumab:1 mg/kg,IV,on days 0, 14, 28, 42, 56;	F1:14/26 F3:91/71 F4:102/95
8	Richard Haynes	2014	United Kingdom	Alemtuzumab:426 Control (Basiliximab):426	554/298	Kidney	Alemtuzumab:52.1±13.3 Control:51.8±13.3	Alemtuzumab:30 mg,IV,on days 0, 1; Basiliximab:20mg,IV, on days 0,4;	F1:31/68 F3:409/413 F4:413/422 F5:136/136
9	Robert J Stratta	2008	United States	Alemtuzumab:28 Control(rATG):18	34/12	SPKT	Alemtuzumab:43.8±7.9 Control:42.9±7.3	Alemtuzumab:30 mg,IV,on days 0, 1; rATG:1.5 mg/kg,IV,on days 0, 1,2;	F1:5/7 F3:22/10 F4:23/18 F5:10/12
10	Nicole A. Pilch	2014	United States	rATG:102 Control (Basiliximab):98	121/79	Kidney	rATG:52±13 Control:52±13	rATG:1.5 mg/kg,IV,on days 0, 1,2,3,4; Basiliximab:20 mg,IV, on days 0,4;	F1:10/8 F3:96/92 F4:99/97 F5:21/10
11	John C Mullen	2014	Canada	Daclizumab:15 Control(rATG):15	23/7	Heart	Daclizumab:58±3 Control:57±3	Daclizumab:2 mg/kg,IV, on day 0;1 mg/kg,IV, on day 4; rATG:1.5 mg/kg,IV,on days 0, 1,2;	F1:0/2 F3:15/15 F4:13/13 F5:10/10
12	P. Jaksch	2010	Austria	Alemtuzumab:30 Control(rATG):30	34/16	Lung	Alemtuzumab:52.3±11.2 Control:49.3±12.6	Alemtuzumab:30 mg,IV,on days 0, 1; rATG:5 mg/kg,on days 0,4;	F1:4/7 F3:30/30 F4:26/29 F5:4/6
13	Gaetano Ciancio	2014	United States	rATG:43 Control (Daclizumab):42	54/31	Kidney	rATG:47.8 Control:49.2	rATG:1mg/kg/day,IV,on days 0-6; Daclizumab:1mg/kg,IV, on day 0,and on weeks 2,4,6,8;	F1:5/8 F3:38/37 F4:39/33 F5:19/15
14	Tieming LU	2011	China	Alemtuzumab:11 Control(rATG):11	9/13	Kidney	Alemtuzuma:38.9± 4.2 Control:40.8± 4.4	Alemtuzuma: 15mg,IV,on days 0,2; rATG:1.5mg/kg,IV,on days 0,2;	F1:2/3 F3:10/9 F4:10/9 F5:4/3
15	Michael J. Hanaway	2011	United States	Alemtuzumab:234 Contro(Basiliximab):171 Control(rATG):69	304/170	Kidney	Alemtuzuma:48.0±13.0 Control (Basiliximab):48.9±13.6 Control(rATG):48.5±11.0	Alemtuzumab:30 mg,IV,on days 0, 1; Basiliximab:20mg,IV, on days 0,4; rATG:1.5mg/Kg,IV,on days 0,2;	F1:12/29/9 F3:218/154/63
16	Aljosˇa Kandus	2010	Slovenia	Basiliximab:107 Control (Daclizumab):105	122/90	Kidney	Basiliximab:48.1±11 Control:49.1±11	Basiliximab:20 mg,IV,on days 0,4; Daclizumab:1 mg/kgIV,on day 0 and on weeks 2, 4, 6, 8;	F1:11/10 F3:101/95 F4:104/102 F5:50/50
17	Christian Noël	2009	France, Belgium	rATG:113 Control (Daclizumab):114	111/116	Kidney	rATG:45.4±10.3 Control:46.9±9.0	rATG:1.25 mg/kg,IV on days 0,1,2,3,4,5,6,7; Daclizumab:1mg/kg,IV,on days 0,14,28,42,56;	F1:17/31 F3:93/98 F4:108/110 F5:53/53
18	Karen L. Hardinger	2009	United States	rATG:142 Control (Basiliximab):137	–	Kidney	rATG: < 50:(46.8%); 50-60:(25.5%); > 60:(27.7%); Control: <50:(46.7%); 50-60:(27.7%); >60:(25.6%);	rATG:1.5 mg/kg,IV,on days 0,1,2,3,4; Basiliximab:20 mg,IV,on days 0,4;	F1:22/35 F3:129/123 F4:136/131
19	Alan C. Farney	2007	United States	Alemtuzumab:113 Control(rATG):109	129/93	SPKT	Alemtuzumab:51±12 Control:49±13	Alemtuzumab:30 mg,IV,on days 0, 1; rATG:1.5 mg/kg,IV,on days 0,1,2,3,4;	F1:16/28 F3:102/96 F4:109/104 F5:8/18
20	Matthew G. Hartwig	2008	United States	rATG:22 Control (Placebo):22		Lung	rATG:47 (20–66); Control:51 (20–64)	rATG:1.5 mg/kg,on days 0,1,2;	F1:6/9 F5:6/9
21	Karen L. Hardinger	2008	United States	rATG:48 Control(hATG):24	46/26	Kidney	rATG:44±14 Control:53±11	rATG:1.5 mg/kg,IV,on days 0,1,2,3,4,5,6; hATG:15 mg/kg,IV,on days 0,1,2,3,4,5,6;	F1:2/6 F3:47/20 F4:36/16 F5:6/8
22	Marcelo Cantarovich	1986	France	ALG:60 Control (SOC):63	88/35	Kidney	ALG:36±9 Control:40±10	ALG:1 mg/kg,IV, on day 0;3 mg/kg on day 1,3,5;	F1:18/47 F3:56/50 F4:38/57 F5:11/6
23	John C. Mullen	2007	Canada	Daclizumab:25 Control(rATG):25	28/22	Lung	Daclizumab:53±3 rATG:52±2	Daclizumab:2 mg/kg IV,on day 0; 1mg/kg IV on day 4; rATG:1.0 mg/kg IV,on days 0,1,2,3,4,5,6,7,8;	F1:13/16 F3:24/24 F4:24/23
24	Marie Françoise Mattei	2004	France	Basiliximab:38 Control(rATG):42	73/7	Heart	Basiliximab:52.7±8.1 Control:49.6±11.6	Basiliximab:20 mg,IV, on days 0,4; rATG:2.5mg/kg/day,IV,on days 0,3,4,5;	F1:19/19 F4:33/33 F5:27/35
25	Michel Carrier	2007	Canada	Basiliximab:17 Control(rATG):18	27/8	Heart	Basiliximab:54±9 Control:54±12	Basiliximab:20 mg on days 0,4; rATG:125 mg,IV, on days 0,1,2	F1:11/15 F4:13/14 F5:3/5
26	Javier Segovia	2005	Spain	Basiliximab:48 Control (OKT3):51	83/16	Heart	Basiliximab:56.5±9.5 Control:55.8±11.2	Basiliximab:20 mg,IV,on days 0,4; OKT3:5mg,IV,on days 0,6;	F1:19/21 F4:45/42 F5:27/39
27	Daniel C. Brennan	2006	United States	rATG:141 Control (Basiliximab):137	161/117	Kidney	rATG:51.3±13.1 Control:49.7±13.0	rATG:1.5 mg/kg,IV,on days 0,1,2,3,4; Basiliximab:20 mg,IV,on days 0,4;	F1:22/35 F3:128/123 F4:135/131 F5:121/103
28	Anantharaman Vathsala	2005	Singapore	Alemtuzumab:20 Control (SOC):10	15/15	Kidney	Alemtuzumab: 37.6 (range:21.2-56.0) Control: 41.1 (range:25.1-54.2)	Alemtuzumab:20mg,IV,on days 0, 1;	F1:5/2 F3:17/10 F4:19/10 F5:9/2
29	Hussein A. Sheashaa	2005	Egypt	Basiliximab:50 Control (SOC):50	85/15	Kidney	Basiliximab:32.98± 9.9 Control:32.58±10.8	Basiliximab:20 mg,IV,on days 0,4;	F1:18/31 F3:50/49 F4:50/48
30	Mandeep R. Mehra	2005	United States	Basiliximab:25 Control (Placebo):31	42/14	Heart	Basiliximab:56.3±9.70 Control:53.4±10.26	Basiliximab:20 mg,IV,on days 0,4;	F1:12/11 F4:22/30 F5:21/23
31	Ray E. Hershberger	2005	United States, Canada, Germany, Sweden	Daclizumab:216 Control (Placebo):218	348/86	Heart	Daclizumab:53.1±11.9 Control:52.4±11.0	Daclizumab 1 mg/kg,IV,on days 0,4,8,22,36,50;	F1:73/101 F3:216/218 F4:195/206 F5:71/80
32	Karen L. Hardinger	2004	United States	rATG:48 Control(hATG):24	45/27	Kidney	rATG:45±3.14 Control:52±3.12	rATG:1.5 mg/kg,IV,on days 0,1,2,3,4,5,6; hATG:15 mg/kg,IV,on days 0,1,2,3,4,5,6;	F1:2/4 F3:34/16 F4:41/17 F5:0/2
33	Joseph G. Lawen	2003	United States	Basiliximab:59 Control (Placebo):64	86/37	Kidney	Basiliximab:45.4 ±13.1 Control:45.9 ±12.1	Basiliximab:20 mg,IV,on days 0,4;	F1:9/17 F3:56/59 F4:59/64 F5:37/45
34	Peter Neuhaus	1998	Europe, Canada, United States	Basiliximab:188 Control (Placebo):193	241/140	Liver	Basiliximab:50.2(20-72) Control:49.0(20-68)	Basiliximab:20 mg,IV,on days 0,4;	F1:66/84 F3:176/178 F4:163/162
35	Hans Sollinger	2001	United States	Basiliximab:70 Control(rATG):65	79/56	Kidney	Basiliximab:44.5±13.7 Control:49.8±11.9	Basiliximab:20 mg,IV,on days 0,4; rATG:1.5mg/kg,IV,on days 0-13;	F1:13/12 F3:55/50 F4:66/63 F5:53/50
36	Ponticelli Claudio	2001	Italy, Europe, Israel, Mexico, South Africa	Basiliximab:168 Control (Placebo):172	227/113	Kidney	Basiliximab:44.2 (18-70) Control:44.2 (18-70)	Basiliximab:20 mg,IV,on days 0,4;	F1:35/60 F3:152/152 F4:166/169 F5:110/113
37	Malcolm V. Brock	2001	United States	OKT3:30 rATG:34 Control (Daclizumab):23	43/44	Lung	OKT3:51±12 rATG:51±11 Daclizumab:52±13	OKT3:5mg/day,IV,on days 0-6; rATG:1.5 mg/kg,IV,on days 0-13; Daclizumab:2 mg/kg IV,on day 0; 1mg/kg IV on day 4;	F1:7/5/1 F3:29/29/22 F4:28/32/21 F5:23/25/10
38	Henrik Ekberg	2000	Sweden	Daclizumab:267 Control (Placebo):268	350/185	Kidney	Daclizumab:46±0.8 Control:47±0.8	Daclizumab:1 mg/kg,IV,on day 0,and on weeks2,4,6,8;	F1:74/116 F3:244/232 F4:263/262 F5:185/193
39	Scott M. Palmer	1999	United States	rATG:22 Control (Placebo):22	23/21	Lung	rATG:47(20-66) Control:51(20-64)	rATG:1.5mg/kg/d,IV,on days 0,1,2;	F1:5/12 F4:14/15 F5:13/9
40	Nashan Björn	1999	Germany	Daclizumab:140 Control (Placebo):133	194/79	Kidney	Daclizumab:44±13 Control:46±12	Daclizumab:1.0 mg/kg,1 mg,IV,on day 0,and on weeks 2,4,6,8;	F1:39/63 F3:124/111 F4:139/125 F5:105/97
41	Daniel C. Brennan	1999	United States	rATG:48 Control(hATG):24	45/27	Kidney	rATG:44±14 Control:52±12	rATG:1.5 mg/kg,IV,on days 0-6; hATG 15 mg/kg,IV,on days 0-6;	F1:2/6 F3:43/21 F4:47/23 F5:27/18
42	Damien Thibaudin	1998	France	rATG:47 Control (Placebo):42	57/31	Kidney	rATG:47±12 Control:46±13	rATG:1.25 mg/kg/d,IV,on days 0-9;	F1:18/27 F3:45/33 F4:44/34
43	F. Lavin	1998	United States, Canada, Sweden	Daclizumab:126 Control (Placebo):134	155/105	Kidney	Daclizumab:47±13 Control:47±13	Daclizumab:1.0 mg/kg,IV,on day 0,and on weeks 2,4,6,8;	F1:28/47 F3:120121 F4:125/131 F5:0/3
44	Diego Cantarovich	1997	France	rATG:25 Control (SOC):25	28/22	SPKT	rATG:43(33-53) Control:39(24-51)	rATG:1.25mg/kg/d,IV,on days 0-9;	F1:9/19 F3:41/38 F4:23/24 F5:15/10
45	Teun van Gelder	1996	Netherlands	Inolimomab:31 Control (OKT3):29	49/11	Heart	Inolimomab:53(14-65) Control:51(24-65)	Inolimomab:10 mg,IV, on days 0-5; OKT3:5 mg,IV,on days 0-5;	F1:5/6 F4:29/29
46	Jan M. Langrehr	1997	Germany	Inolimomab:39 Control(rATG):41	52/28	Liver	Inolimomab:42.5±4.3 Control:38.6±3.7	Inolimomab:10 mg/day,IV,on days 0-5; rATG:5mg/kg/day,IV,on days 0-5;	F1:18/8 F3:38/40 F4:33/36
47	Hourmant Maryvonne	1996	France	Odulimomab:52 Control(rATG):49	83/28	Kidney	Odulimomab:46±11 Control:45±11	Odulimomab:30 mg,IV,on day 0,and 15 mg,on days 1-8; rATG:1.25mg/kg,IV,on days 0-9;	F1:24/19 F4:50/49
48	Teun van Gelder	1995	Netherlands	Inolimomab:30 Control (Placebo):30	37/23	Kidney	Inolimomab:45(19-65) Control:43(22-60)	Inolimomab:10 mg/day,IV,on days 0-5;	F1:0/7 F3:28/28 F4:30/27
49	Douglas W.Hanto	1994	United States	ALG:59 Control (OKT3):58	71/46	Kidney	ALG:43±11 Control:44±11	OKT3:5 mg,IV,on days 0-5; ALG:1mg/kg,IV, on day 0;3 mg/kg on day 1,3,5;	F1:2/1 F3:49/48 F4:56/57
50	C. Vela	1993	France	OKT3:15 Control (ALG):23	16 / 22	Kidney	OKT3:45±2 Control:48±2	OKT3:5 mg,IV,on days 0-9 ; ALG:1 mg/kg,IV, on day 0;3 mg/kg on day 1,3,5;	F1:8/12 F3:9/19 F4:14/22 F5:5/12
51	Douglas J. Norman	1993	United States	OKT3:105 Control (SOC):102	131/76	Kidney	OKT3:43 (12-73) Control:40(10-66)	OKT3:5 mg,IV,on days 0-5;	F1:54/67 F3:95/84
52	Peter S. Macdonald	1990	Austria	OKT3:20 Control(rATG):21	35/6	Heart	OKT3:48.7(30-65) Control:44.1(18-59)	OKT3:5 mg,IV,on days 0-5;	F1:18/21 F4:17/17
53	S. Beaudreuil	2006	France	Lo-tact-1:20 Control (rATG):20	23/17	Kidney	Lo-tact-1:42.1±12.4 Control:39.3±11.3	Lo-tact-1:10 mg/day,IV,on days 0-13; ATG:1.5mg/day,IV,on days 0-13;	F1:10/9
54	Josep M. Grino	1992	Spain	ALG:68 Control (OKT3):72	116/24	Kidney	ALG:42.6±13 Control:39±11	ALG:1.5mg/kg,IV,on days 0-4; OKT3:5mg,on days 0-4;	F1:10/14 F3:56/67 F4:66/71
55	R. L. Kirkman	1991	United States	Daclizumab:40 Control (SOC):40	49/31	Kidney	Daclizumab:43.8(20-61) Control:44.0(16-63)	Daclizumab:1.0 mg/kg,1 mg,IV,on day 0,and on weeks 2,4,6,8;	F1:14/24 F3:33/34 F4:35/38
56	Douglas J. Norman	1987	United States	OKT3:34 Control (SOC):38	42/38	Kidney	–	OKT3:5mg/d,IV,on days 0-4;	F1:6/19 F3:31/29 F4:34/38
57	Raymond Reding	1993	Belgium	Lo-tact-1:35 OKT3:37 Control (SOC):28	57/43	Liver	Lo-tact-1:46(25-62) OKT3:47(26-57) Control:45(25-58)	Lo-tact-1:10 mg/day,IV,on days 0-13; OKT3:5mg/d,IV,on days 0-4;	F1:32/30/27 F5:14/18/11
58	A. H. M. M. Balk	1992	Netherlands	ALG:28 Control (OKT3):27	55/10	Heart	ALG:45(18-61)Control:48(15-62)	ALG:1.5mg/kg,IV,on days 0-4; OKT3:5mg/d,IV,on days 0-4;	F1:5/9 F4:27/26 F6:20/27
59	S.V. McDiarmid	1991	United States	OKT3:25 Control (SOC):27	–	Liver	OKT3:44.8 Control:46.1	OKT3:5mg/d,IV,on days 0-4;	F1:9/7 F3:19/21 F4:10/19 F5:18/11
60	H. A. Sheashaa	2008	Egypt	Basiliximab:50 Control (SOC):50	85/15	Kidney	Basiliximab:32.9±9.9 Control:32.5±10.8	Basiliximab:20 mg,IV,on days 0,4;	F1:18/31 F3:41/40 F4:46/46
61	Henry ML	2001	United States	OKT3:54 Control (SOC):49	70/33	Kidney	OKT3:45(16-74) Control:49(16-76)	OKT3:5mg/d,IV,on days 0-4;	F1:6/13 F3:54/48 F4:54/46 F5:11/8
62	Gaetano Ciancio	2008	USA	rATG:30 Control (Alemtuzumab):30 Control (Daclizumab):30	56/34	Kidney	rATG:49.3±2.5 Alemtuzumab:50.2±2.1 Daclizumab:49.9±2.4	rATG:1.25mg/kg/d,IV,on days 0-9; Alemtuzumab:30 mg,IV,on days 0, 1; Daclizumab:2 mg/kg IV,on day 0; 1mg/kg IV on day 4;	F1:7/2/2
63	Maximilian Schmeding	2007	Germany	Basiliximab:51 Control (SOC):48	54/45	Liver	Basiliximab:49.4 Control:49.6	rATG:1.25mg/kg/d,IV,on days 0-9;	F1:19/15 F3:49/48 F5:30/28
64	Min Jeong Kim	2008	Switzerland	rATG:11 Control (Daclizumab):11	6/16	Kidney	rATG:52(39–68) Control:51(34–60)	rATG:1.25mg/kg/d,IV,on days 0-9; Daclizumab:2 mg/kg IV,on day 0; 1mg/kg IV on day 4;	F1:4/5 F3:11/11 F4:10/10 F5:9/11
65	J. Tan	2005	China	Basiliximab:36 Control (SOC):20	19/37	Kidney	Basiliximab:50±11.6 Control:45±9.3	Basiliximab:20 mg,IV,on days 0,4;	F1:4/10 F3:36/18 F4:36/18 F5:0/2
66	R.J. Stratta	2009	United States	Alemtuzumab:28 Control(rATG):18	34/12	Kidney and Pancreas	Alemtuzumab:43.8±7.9 Control:42.9±7.3	Alemtuzumab: 15mg,IV,on days 0,2; rATG:1.25mg/kg/d,IV,on days 0-9;	F1:5/7 F4:23/18 F5:11/12
67	A. Duzova	2002	Turkey	Basiliximab:20 Control (SOC):23	25 /18	Kidney	Basiliximab:14.9±3.6 Control:15.3±4.2	Basiliximab:20 mg,IV,on days 0,4;	F1:1/6 F3:20/20 F4:20/22
68	Hussein A. Sheashaa	2011	Egypt	Basiliximab:50 Control (SOC):50	85/15	Kidney	Basiliximab:32.9±9.3 Control:32.5±10.8	Basiliximab:20 mg,IV,on days 0,4;	F1:18/31 F3:38/34 F4:46/46

M/F, Male/Female; SPKT, Simultaneous pancreas-kidney transplantation.

F1: Rejection rate.

F3: Graft survival rate.

F4: Overall survival rate.

F5: Infection rate.

### 3.3 Intervention network

**Figure 2:**
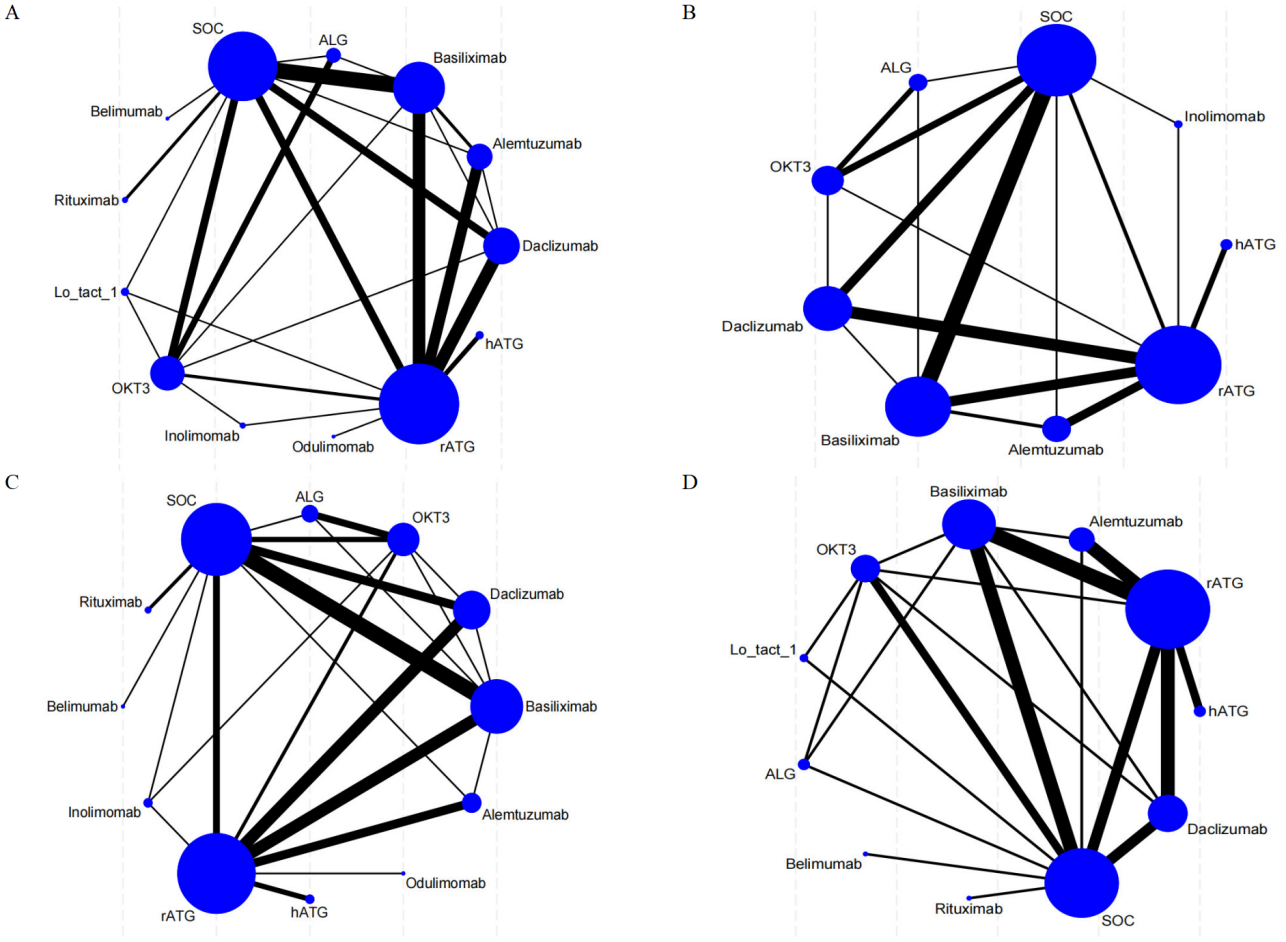
Network meta-analysis maps illustrate the studies assessing the efficacy and safety of induction therapy in SOT concerning **(A)** rejection rate, **(B)** graft survival rate, **(C)** overall survival rate, and **(D)** infection rate. The size of the nodes reflects the number of participants within each intervention, while the thickness of the lines connecting the interventions indicates the number of studies associated with each comparison.

Direct comparisons among therapies are visualized in **Figure 2**. Nodes represent interventions (size proportional to sample size), and edges denote direct comparisons (thickness indicating study count).

### 3.4 ROB assessment

**Figure 3:**
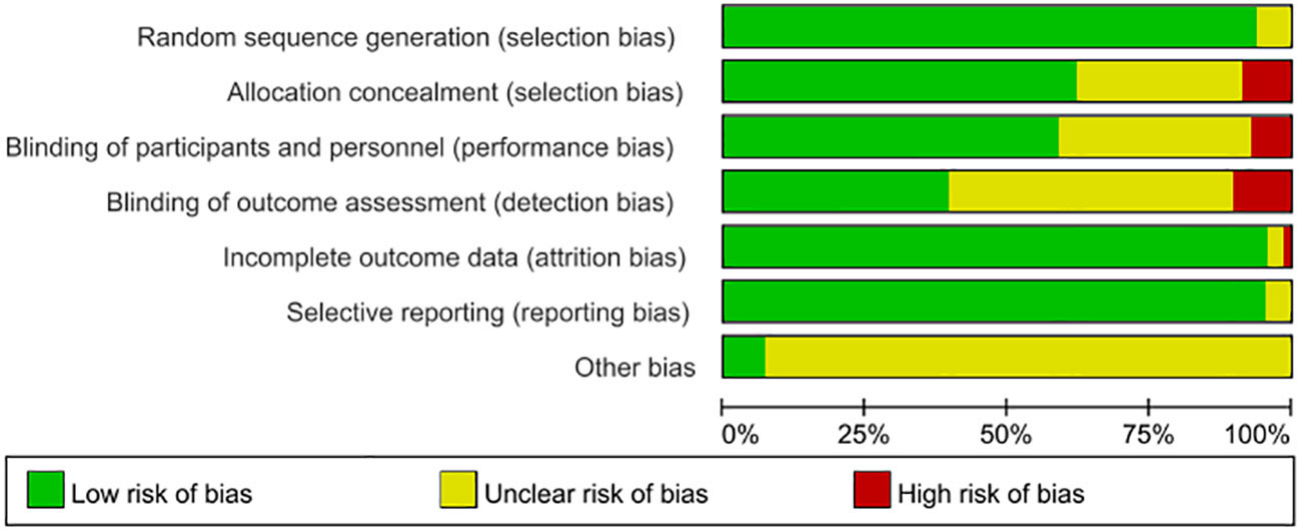
Bias risk assessment chart.

Among 68 studies, 60 described explicit randomization methods, while 64 reported allocation concealment. Double-blinding was implemented in 42 trials, and 29 blinded outcome assessors. Eleven studies documented participant attrition with reasons. All studies exhibited low selective reporting risk. ROB evaluations are detailed in **Figure 3**.

### 3.5 NMA outcomes

#### 3.5.1 Rejection rate

**Figure 4:**
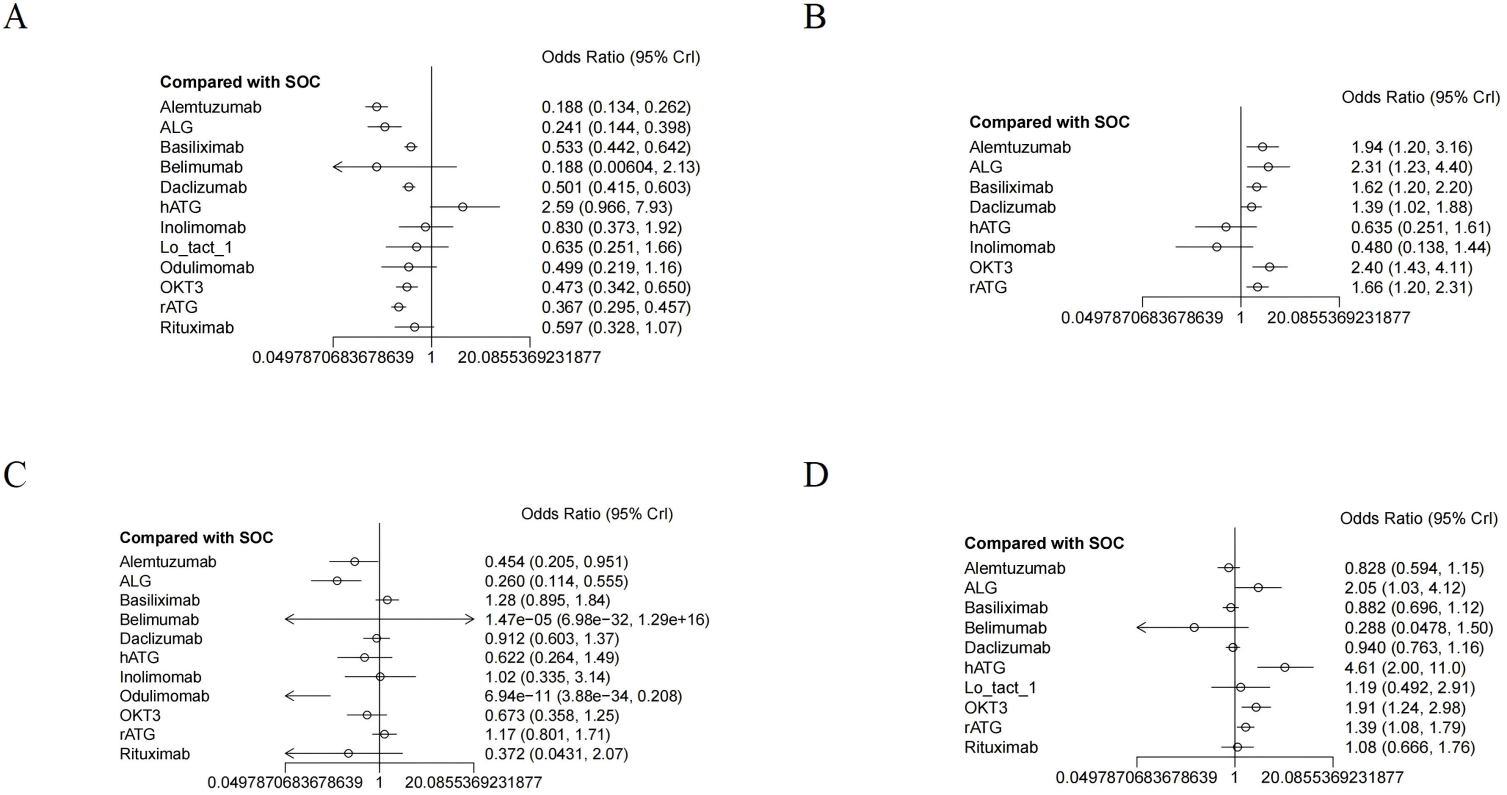
The forest plot illustrates the studies assessing the efficacy and safety of induction therapy in SOT concerning **(A)** rejection rate, **(B)** graft survival rate, **(C)** overall survival rate, and **(D)** infection rate.

**Figure 5:**
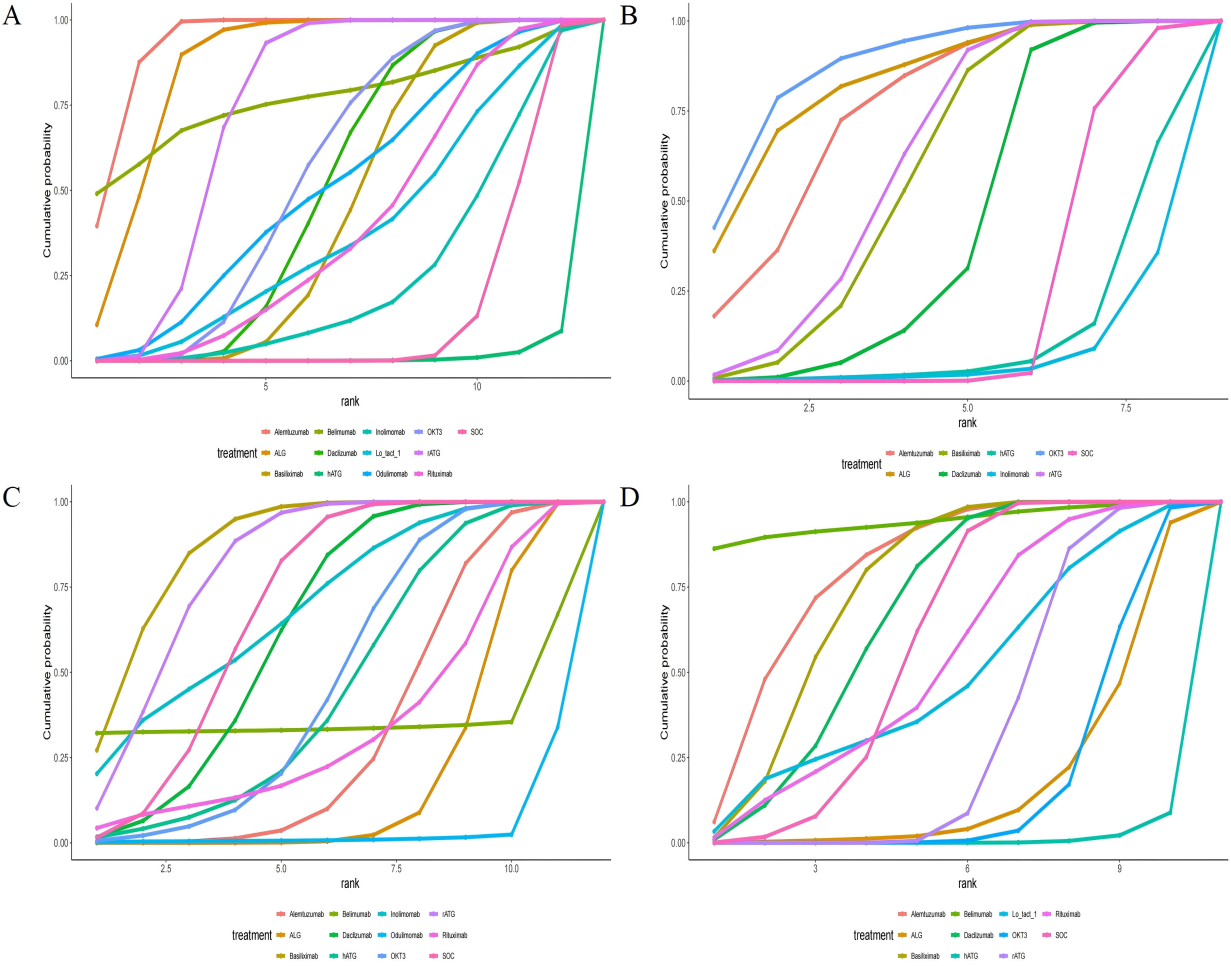
Area under the cumulative probability curve. **(A)** rejection rate, **(B)** graft survival rate, **(C)** overall survival rate, **(D)** infection rate.

All included RCTs reported rejection rates. Network meta-analysis (NMA) revealed significant reductions in rejection rates with alemtuzumab, ALG, belimumab, rATG, OKT3, daclizumab, odulimomab, basiliximab, rituximab, Lo-tact-1, and inolimomab compared to controls, whereas hATG showed no superiority (**Figure 4A**, **Table 2**). Surface Under the Cumulative Ranking Area (SUCRA) scores ranked interventions as follows: alemtuzumab (93.9%) > ALG (87.0%) > belimumab (77.0%) > rATG (73.6%) > OKT3 (55.4%) > daclizumab (51.0%) > odulimomab (50.8%) > basiliximab (44.5%) > rituximab (39.8%) > Lo-tact-1 (38.0%) > inolimomab (24.3%) > SOC (13.8%) > hATG (0.1%) (**Table 3**). Alemtuzumab emerged as the most effective therapy for reducing rejection in SOT recipients undergoing induction therapy (cumulative ranking: **Figure 5A**).

**Table 2:** League table.

OR 95% CI (rejection rate)												
Alemtuzumab												
0.78 (0.43, 1.43)*	ALG											
0.35 (0.26, 0.47)*	0.45 (0.26, 0.76)*	Basiliximab										
1 (0.09, 31.21)*	1.29 (0.11, 40.68)*	2.84 (0.25, 88.09)*	Belimumab									
0.38 (0.26, 0.54)*	0.48 (0.28, 0.82)*	1.07 (0.84, 1.35)*	0.38 (0.01, 4.25)*	Daclizumab								
0.07 (0.02, 0.2)*	0.09 (0.03, 0.28)*	0.21 (0.07, 0.55)*	0.07 (0, 1.01)	0.19 (0.06, 0.53)*	hATG							
0.23 (0.09, 0.52)*	0.29 (0.11, 0.74)*	0.64 (0.28, 1.43)*	0.22 (0.01, 2.92)*	0.6 (0.26, 1.36)*	3.12 (0.87, 11.94)*	Inolimomab						
0.29 (0.11, 0.77)*	0.38 (0.13, 1.06)*	0.83 (0.31, 2.12)*	0.29 (0.01, 3.93)*	0.78 (0.29, 2)*	4.03 (1.02, 16.75)*	1.3 (0.37, 4.39)*	Lo-tact-1					
0.38 (0.16, 0.88)*	0.48 (0.18, 1.27)*	1.07 (0.47, 2.42)*	0.37 (0.01, 4.9)*	1 (0.43, 2.31)*	5.17 (1.45, 19.84)*	1.66 (0.53, 5.19)*	1.29 (0.37, 4.49)*	Odulimomab				
0.4 (0.25, 0.62)*	0.51 (0.31, 0.83)*	1.13 (0.8, 1.6)*	0.4 (0.01, 4.6)*	1.06 (0.74, 1.53)*	5.45 (1.94, 17.2)*	1.76 (0.78, 4.07)*	1.36 (0.53, 3.63)*	1.06 (0.44, 2.57)*	OKT3			
0.51 (0.37, 0.69)*	0.66 (0.38, 1.12)*	1.45 (1.2, 1.76)*	0.51 (0.02, 5.82)*	1.36 (1.07, 1.74)*	6.98 (2.66, 20.87)*	2.25 (1.03, 5.08)*	1.74 (0.69, 4.61)*	1.36 (0.61, 3.04)*	1.28 (0.89, 1.85)*	rATG		
0.32 (0.16, 0.62)*	0.4 (0.18, 0.88)*	0.9 (0.49, 1.67)*	0.31 (0.01, 3.82)*	0.84 (0.46, 1.57)*	4.34 (1.36, 15.25)*	1.4 (0.51, 3.85)*	1.08 (0.36, 3.37)*	0.84 (0.31, 2.36)*	0.79 (0.4, 1.56)*	0.62 (0.33, 1.17)*	Rituximab	
0.19 (0.13, 0.26)*	0.24 (0.14, 0.4)*	0.53 (0.44, 0.64)*	0.19 (0.01, 2.13)*	0.5 (0.42, 0.6)*	2.57 (0.95, 7.82)*	0.83 (0.37, 1.9)*	0.64 (0.25, 1.7)*	0.5 (0.22, 1.15)*	0.47 (0.34, 0.65)*	0.37 (0.29, 0.46)*	0.6 (0.33, 1.07)*	SOC

OR 95% CI (graft survival rate)												
Alemtuzumab												
0.84 (0.38, 1.83)*	ALG											
1.19 (0.79, 1.81)*	1.42 (0.72, 2.84)*	Basiliximab										
1.4 (0.85, 2.32)*	1.67 (0.84, 3.36)*	1.17 (0.83, 1.67)*		Daclizumab								
3.06 (1.16, 8)*	3.64 (1.19, 11.06)*	2.56 (1.03, 6.35)*		2.18 (0.86, 5.54)*	hATG							
4.03 (1.28, 14.71)*	4.84 (1.36, 19.31)*	3.38 (1.13, 11.79)*		2.89 (0.95, 10.16)*	1.33 (0.33, 5.93)*	Inolimomab						
0.81 (0.4, 1.61)*	0.96 (0.55, 1.67)*	0.68 (0.37, 1.21)*		0.58 (0.32, 1.04)*	0.26 (0.09, 0.76)*	0.2 (0.05, 0.67)*	OKT3					
1.16 (0.77, 1.78)*	1.39 (0.69, 2.8)*	0.98 (0.75, 1.28)*		0.83 (0.6, 1.16)*	0.38 (0.16, 0.91)*	0.29 (0.08, 0.85)*	1.45 (0.8, 2.66)*			rATG		
1.94 (1.2, 3.16)*	2.31 (1.23, 4.4)*	1.62 (1.2, 2.2)*		1.39 (1.02, 1.88)*	0.64 (0.25, 1.61)*	0.48 (0.14, 1.44)*	2.4 (1.43, 4.11)*			1.66 (1.2, 2.31)*		SOC

OR 95% CI (overall survival rate)												
Alemtuzumab												
2.11 (0.7, 6.5)*	ALG											
0.41 (0.19, 0.83)*	0.19 (0.08, 0.45)*	Basiliximab										
22473.55 (0, 8.94e+28)	10551.26 (0, 4.34e+28)	54709.18 (0, 2.27e+29)		Belimumab								
0.59 (0.25, 1.33)*	0.28 (0.11, 0.68)*	1.44 (0.88, 2.38)*		0 (0, 1.31e+15)	Daclizumab							
0.84 (0.28, 2.4)*	0.4 (0.12, 1.27)*	2.06 (0.86, 4.84)*		0 (0, 1.90e+15)	1.42 (0.58, 3.49)*	hATG						
0.5 (0.14, 1.83)*	0.24 (0.06, 0.93)*	1.23 (0.4, 3.81)*		0 (0, 1.19e+15)	0.85 (0.27, 2.7)*	0.6 (0.16, 2.29)*	Inolimomab					
2.78e+9 (2.28, 1.83e+31)*	1.31e+9 (1.07, 8.32e+30)*	6776429723.69 (5.66, 4.48e+31)*		7.11e+5 (0, 1.88e+34)	4.71e+9 (3.95, 3.06e+31)*	3.33e+9 (2.74, 2.19e+31)*	5.48e+9 (4.36, 3.53e+31)*	Odulimomab				
0.66 (0.23, 1.86)*	0.31 (0.11, 0.82)*	1.61 (0.73, 3.58)*		0 (0, 1.50e+15)	1.12 (0.47, 2.61)*	0.78 (0.26, 2.38)*	1.31 (0.37, 4.66)*	0 (0, 0.29)	OKT3			
0.45 (0.21, 0.91)*	0.21 (0.08, 0.5)*	1.09 (0.76, 1.59)*		0 (0, 1.03e+15)	0.76 (0.48, 1.18)*	0.53 (0.25, 1.16)*	0.89 (0.3, 2.63)*	0 (0, 0.19)	0.68 (0.31, 1.5)*	rATG		
0.55 (0.01, 24.23)*	0.26 (0.01, 11.45)*	1.35 (0.03, 56.24)*		0 (0, 1.48e+15)	0.94 (0.02, 39.55)*	0.66 (0.02, 29.17)*	1.09 (0.02, 52.54)*	0 (0, 0.4)	0.84 (0.02, 36.84)*	1.24 (0.03, 51.69)*	Rituximab	
0.53 (0.24, 1.15)*	0.25 (0.11, 0.56)*	1.3 (0.88, 1.94)*		0 (0, 1.17e+15)	0.9 (0.59, 1.37)*	0.63 (0.27, 1.52)*	1.05 (0.34, 3.23)*	0 (0, 0.23)	0.81 (0.36, 1.83)*	1.19 (0.8, 1.77)*	0.96 (0.02, 38.24)*	SOC

OR 95% CI (infection rate)												
Alemtuzumab												
0.41 (0.19, 0.84)*	ALG											
0.94 (0.73, 1.21)*	2.32 (1.16, 4.71)*	Basiliximab										
2.87 (0.53, 17.91)*	7.12 (1.19, 48.79)*	3.06 (0.58, 18.81)*		Belimumab								
0.88 (0.62, 1.25)*	2.18 (1.07, 4.46)*	0.94 (0.72, 1.22)*		0.31 (0.05, 1.62)*	Daclizumab							
0.18 (0.07, 0.42)*	0.44 (0.15, 1.3)*	0.19 (0.08, 0.43)*		0.06 (0.01, 0.4)*	0.2 (0.09, 0.47)*	hATG						
0.69 (0.27, 1.78)*	1.71 (0.57, 5.17)*	0.74 (0.3, 1.83)*		0.24 (0.03, 1.57)*	0.79 (0.32, 1.95)*	3.87 (1.15, 13.23)*	Lo-tact-1					
0.43 (0.26, 0.72)*	1.07 (0.51, 2.27)*	0.46 (0.29, 0.72)*		0.15 (0.02, 0.83)*	0.49 (0.31, 0.78)*	2.41 (0.96, 6.22)*	0.63 (0.26, 1.47)*	OKT3				
0.6 (0.44, 0.8)*	1.47 (0.72, 3.03)*	0.63 (0.51, 0.78)*		0.21 (0.03, 1.1)*	0.68 (0.52, 0.88)*	3.31 (1.5, 7.61)*	0.86 (0.35, 2.14)*	1.37 (0.87, 2.21)*	rATG			
0.77 (0.42, 1.38)*	1.89 (0.81, 4.43)*	0.82 (0.47, 1.4)*		0.27 (0.04, 1.49)*	0.87 (0.51, 1.48)*	4.26 (1.62, 11.5)*	1.1 (0.4, 3.04)*	1.77 (0.92, 3.4)*	1.29 (0.74, 2.22)*		Rituximab	
0.83 (0.59, 1.15)*	2.05 (1.03, 4.12)*	0.88 (0.7, 1.12)*		0.29 (0.05, 1.5)*	0.94 (0.76, 1.16)*	4.61 (2, 11)*	1.19 (0.49, 2.91)*	1.91 (1.24, 2.98)*	1.39 (1.08, 1.79)*		1.08 (0.67, 1.76)*	SOC

*MEANS *p*<0.05.

**Table 3:** SUCRA ranking.

Treatment	Rejection rate (%)	Graft survival rate (%)	Overall survival rate (%)	Infection rate (%)
Alemtuzumab	93.9	75.6	33.8	80.0
ALG	87.0	83.5	20.5	18.1
Basiliximab	44.5	58.2	88.0	74.5
Belimumab	77.0	–	36.5	94.4
Daclizumab	51.0	42.9	63.8	67.3
hATG	0.1	11.7	46.7	1.2
Inolimomab	24.3	6.6	70.3	–
Lo-tact-1	38.0	–	–	49.2
Odulimomab	50.8	–	3.9	–
OKT3	55.4	87.9	48.6	18.3
rATG	73.6	61.7	82.1	33.6
Rituximab	39.8	–	35.7	54.4
SOC	13.8	22.0	70.2	58.8

#### 3.5.2 Graft survival rate

Fifty RCTs evaluated graft survival. NMA demonstrated superior outcomes with OKT3, ALG, alemtuzumab, rATG, basiliximab, and daclizumab versus controls, while hATG and inolimomab lacked significant benefits (**Figure 4B**, **Table 2**). SUCRA rankings were: OKT3 (87.9%) > ALG (83.5%) > alemtuzumab (75.6%) > rATG (61.7%) > basiliximab (58.2%) > daclizumab (42.9%) > SOC (22.0%) > hATG (11.7%) > inolimomab (6.6%) (**Table 3**). OKT3 ranked highest for improving graft survival (cumulative ranking: **Figure 5B**).

#### 3.5.3 Overall survival rate

Sixty-one RCTs assessed overall survival. Basiliximab, rATG, and inolimomab significantly increased survival rates compared to controls, whereas daclizumab, OKT3, hATG, belimumab, rituximab, alemtuzumab, ALG, and odulimomab showed no advantage (**Figure 4C**, **Table 2**). SUCRA scores prioritized basiliximab (88.0%) > rATG (82.1%) > inolimomab (70.3%) > SOC (70.2%) > daclizumab (63.8%) (**Table 3**). Basiliximab was identified as the most effective for enhancing overall survival (cumulative ranking: **Figure 5C**).

#### 3.5.4 Infection rate

Forty-four RCTs evaluated infections. Belimumab, alemtuzumab, basiliximab, and daclizumab significantly reduced infection rates versus controls, while rituximab, Lo-tact-1, rATG, OKT3, ALG, and hATG exhibited higher incidences (**Figure 4D**, **Table 2**). SUCRA rankings were: belimumab (94.4%) > alemtuzumab (80.0%) > basiliximab (74.5%) > daclizumab (67.3%) > SOC (58.8%); conversely, rituximab (54.4%), Lo-tact-1 (49.2%), rATG (33.6%), OKT3 (18.3%), ALG (18.1%), and hATG (1.2%) underperformed (**Table 3**). Belimumab demonstrated the greatest efficacy in reducing infections (cumulative ranking: **Figure 5D**).

### 3.6 PB assessment

**Figure 6:**
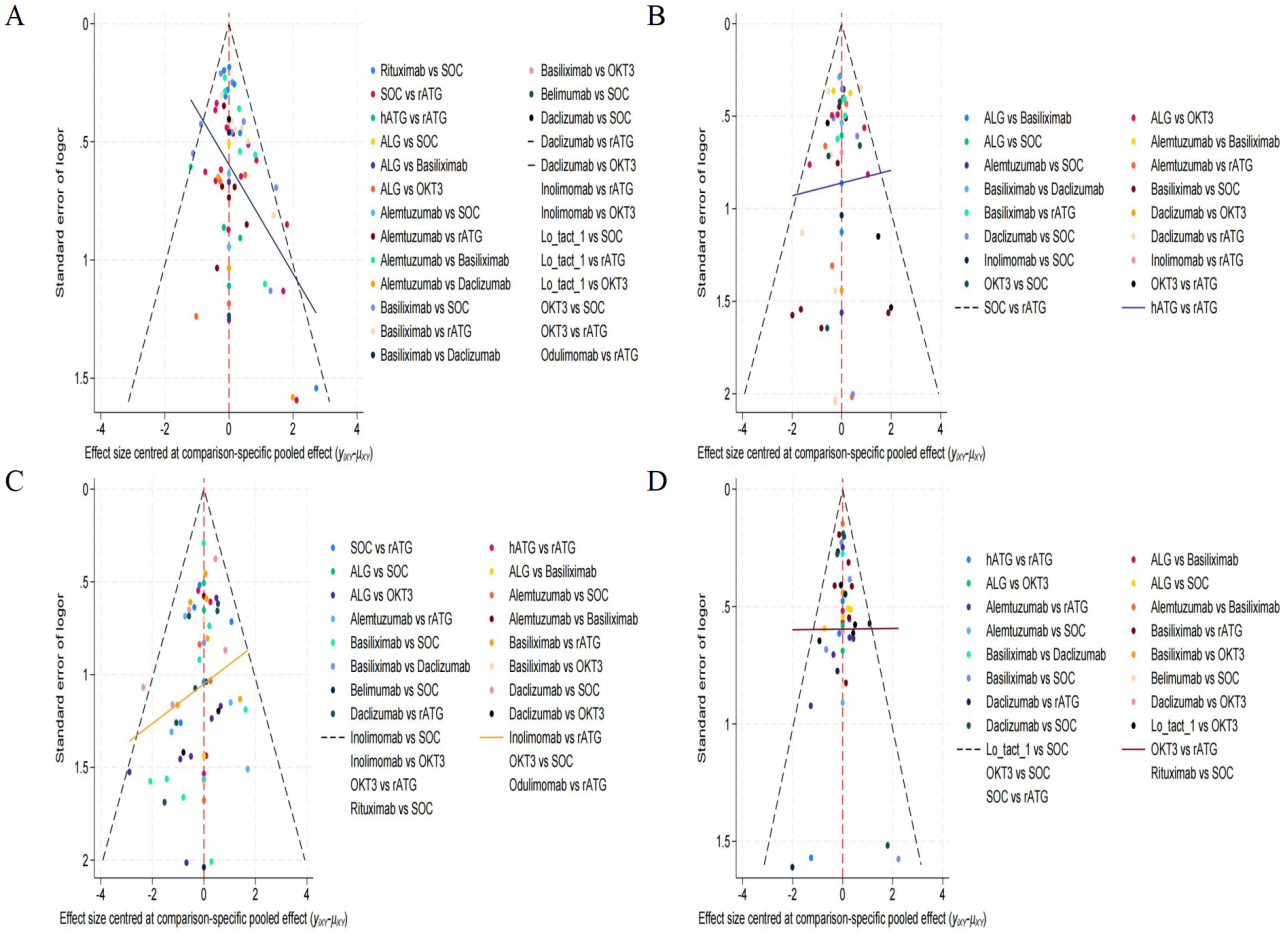
Funnel plot for evaluating publication bias. **(A)** rejection rate, **(B)** graft survival rate, **(C)** overall survival rate, **(D)** infection rate.

The observed asymmetry within the funnel plot presented in **Figure 6**, which constitutes a key component of our publication bias assessment, demonstrates the dispersion of numerous outlying data points positioned beyond the established funnel boundaries. This deviation is particularly pronounced within the lower contour region, a pattern consistent with Egger’s regression results (p<0.05). Such distributional irregularities substantiate the probable existence of small-study effects alongside systematic publication bias. Consequently, the preliminary nature of these findings necessitates their interpretation with substantial scientific caution due to inherent methodological limitations.

## 4 Discussion

### 4.1 Mechanisms, clinical applications, and future directions of monoclonal antibody induction therapies

**Figure 7:**
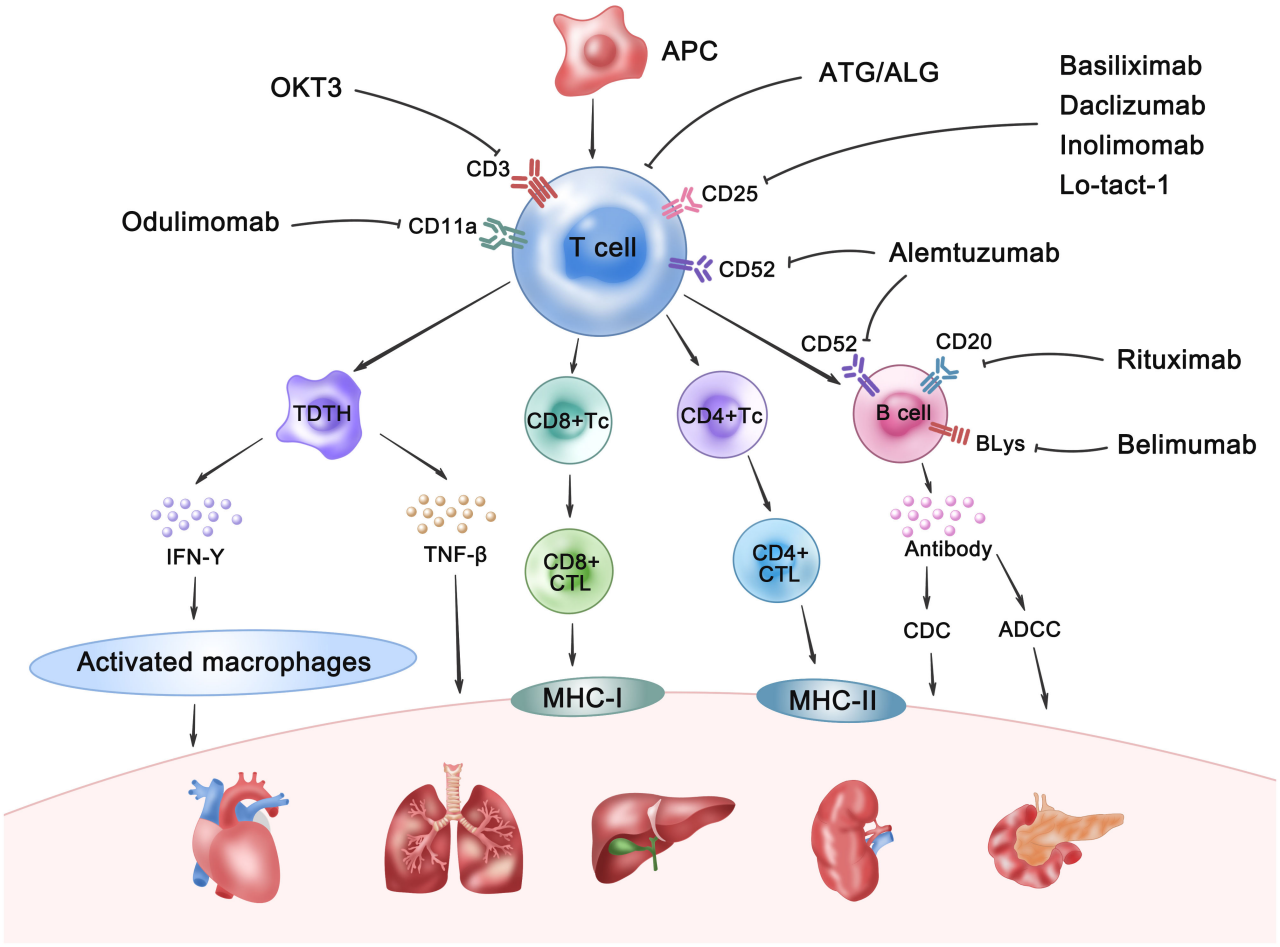
The mechanisms of allograft rejection and the therapeutic targets for its prevention. APC, Antigen-presenting cell; TDTH, Delayed-type hypersensitivity T cell; Tc, T cytotoxic cell; CD4+/CD8+CTL, CD4+/CD8+ cytotoxic T lymphocyte; IFN-γ, Interferon-γ; TNF-β, Tumor Necrosis Factor-β; CDC, Complement-dependent cytotoxicity; ADCC: Antibody-dependent cell-mediated cytotoxicity; BlyS, Anti-B-lymphocyte stimulator.

Historically, polyclonal antibodies such as ATG and ALG were the mainstay of immunosuppressive therapy in pre-transplantation. Their mechanism of action involved antibody-dependent cell-mediated cytotoxicity, complement-dependent cytotoxicity, and phagocytosis by reticuloendothelial cells in the liver, spleen, and lungs. This broad reactivity resulted in efficient elimination of T cells within the thymus and the transplanted organ. However, the xenogeneic nature of these polyclonal antibodies resulted in significant adverse effects, including cytokine release syndrome, serum sickness, anaphylaxis, and increased infection susceptibility (16). This led to their gradual displacement by monoclonal antibodies (17). At present, the advent of monoclonal antibody technology has ushered in a new era of pre-transplant induction therapy, characterized by more specific and effective agents. These monoclonal antibodies target specific lymphocyte subsets or cytokines, enabling more precise immunosuppression and reduced non-specific side effects. Commonly used examples include anti-CD3 (e.g., OKT3), anti-CD25 (e.g., basiliximab, daclizumab, Inolimomab, and Lo-tact-1), and anti-CD52 (e.g., alemtuzumab) monoclonal antibodies, which target T cells, as well as antibodies targeting adhesion molecules and chemokine receptors. Anti-CD20 (e.g., rituximab) and anti-B-lymphocyte stimulator (BLyS; e.g., belimumab) are also utilized. The mechanisms of allograft rejection and the therapeutic targets for its prevention are presented in **Figure 7**. Future advancements in transplant rejection prevention will focus on several key strategies: First, the development of more precisely targeted therapies utilizing monoclonal antibodies to specifically target cellular subsets or signaling pathways crucial in allograft rejection, thereby minimizing off-target effects and adverse events. Second, the implementation of combination therapies synergistically employing monoclonal antibodies with other immunosuppressants, cellular therapies, or gene therapies to enhance efficacy and overcome monotherapy limitations. Third, the use of biomarker-guided therapy, employing predictive biomarkers to personalize immunosuppressant administration, including monoclonal antibodies, based on individual patient risk profiles. Finally, the induction of immunological tolerance, which necessitates further research into tolerance mechanisms and the identification of novel therapeutic targets and strategies (18). The widespread implementation of immunoinduction therapy has significantly enhanced the short-term overall survival rates of solid organ grafts (19). However, a consensus regarding the most effective induction protocol remains elusive. Graft rejection profoundly affects both the functionality of transplanted organs and the survival of recipients (20). Therefore, enhancing graft survival is a pivotal objective in patients undergoing SOT. Despite this, numerous studies have failed to adequately differentiate between the various treatments, thereby limiting clinical applicability.

### 4.2 Efficacy-safety profiles of induction agents via NMA

This NMA comprehensively evaluates the efficacy and safety profiles of various induction therapies in SOT recipients. The findings reveal that the agents demonstrate varying performance characteristics across different critical clinical endpoints: alemtuzumab was the most effective treatment for reducing the rejection rate; basiliximab is acknowledged to be a significant treatment for improving overall survival; belimumab has been recognized for its potential to reduce infection episodes. OKT3 has been identified as the primary treatment for enhancing graft survival. These findings suggest that the selection of induction agents should be determined by weighing multiple therapeutic objectives and prioritizing them according to individualized patient factors. Although our study found that OKT3 as induction therapy significantly enhances graft survival, it has been supplanted by newer pharmacological agents due to the high incidence of adverse events, and will not be further discussed.

### 4.3 Mechanisms of alemtuzumab-mediated immunosuppression

Alemtuzumab targets CD52, which exerts significant immunomodulatory effects by inducing depletion and subsequent repopulation of lymphocytes, resulting in alterations in the number, proportion, and characteristics of specific lymphocyte subsets after treatment. Notably, the immunophenotypic alterations were characterized by elevated frequencies of Treg subsets and expanded populations of memory T and B lymphocytes (21). Zwan et al. (22) compared the efficacy of alemtuzumab and rATG for renal transplantation rejection. They found that alemtuzumab may serve as a viable alternative for patients with severe acute renal transplantation rejection. Furukawa et al. (23) conducted a study comparing belatacept and alemtuzumab and discovered that the alemtuzumab group experienced a significantly reduced incidence of acute rejection events. Similarly, Hanaway et al. (24) and co-authors found in a prospective RCT that compared the SOC and rATG groups, alemtuzumab markedly decreased the proportion of acute rejection among renal transplantation recipients within the first year post-surgery. Nonetheless, they noted that in high-risk groups, no significant difference was found between alemtuzumab and rATG in terms of the reduction in rejection rates. Jaksch et al. (25) conducted a prospective RCT to compare the efficacy of alemtuzumab with rATG in lung transplantation induction therapy. They found that alemtuzumab significantly reduced the occurrence of high-grade rejection events. Although alemtuzumab significantly reduces the incidence of acute rejection, this advantage fails to translate into long-term survival benefits (26). The likely reason is that its prolonged and profound immune erosion may delay immune reconstitution and lead to a significantly elevated risk of long-term opportunistic infections, which is probably a key factor underlying its failure to achieve optimal graft and patient survival outcomes (26). This underscores that rejection control is not the sole determinant of long-term success. Non-rejection-related causes of death, including infections, cardiovascular events, and malignancies, contribute disproportionately to mortality in patients treated with the potent depleting agent alemtuzumab, thereby attenuating the potential survival benefit conferred by its superior rejection control (27). This observation carries implications for clinical decision-making: alemtuzumab could be considered a preferred induction agent for highly immunogenic organ transplants (e.g., kidney, heart, lung, pancreas), particularly in recipients with a high degree of HLA mismatching and inherently low infection risk. However, clinicians must diligently manage patients’ short-term infection risks and prioritize long-term survival.​ In conclusion, a substantial body of research has substantiated alemtuzumab in reducing rejection, which is consistent with our findings.

### 4.4 Mechanisms of basiliximab-mediated immunosuppression

Interleukin-2 (IL-2) plays a critical role in the pathogenesis of acute rejection by activating donor cytotoxic T lymphocytes that specifically target receptor antigens. Consequently, blockade of IL-2 receptors has emerged as a crucial strategy in the induction therapy of organ transplantation (28). Basiliximab, a chimeric monoclonal antibody (IgG1κ) composed of murine and human components, selectively antagonizes the alpha chain of IL-2 receptor (CD25 antigen). Activated T lymphocytes demonstrate a high affinity for IL-2, enabling basiliximab to bind with remarkable specificity to the CD25 antigen, thereby significantly reducing the CD25 levels (29). This reduction influences the interactions between CD4/CD25/CD127-Foxp3/Tregs, effectively disrupting the signaling pathways that facilitate T cell proliferation (30). Importantly, the pharmacological mechanism of basiliximab is fundamentally distinct from that of polyclonal antibodies, such as ATG, because it does not induce lymphocyte depletion. This targeted mechanism, which preserves the functionality of alternative effector pathways, has a relatively minor impact on susceptibility to post-transplant infections associated with the use of anti-CD25 monoclonal antibodies as induction therapy, particularly compared with ATG (31). Furthermore, basiliximab offers substantial advantages in enhancing recipient overall survival rates, improving graft survival, and reducing rejection (32), establishing it as a preferred option in certain centers to prevent acute rejection during SOT (33). Mo et al. (34) et al. conducted a multicenter analysis involving over 900 recipients, demonstrating that basiliximab is both safe and effective for steroid-resistant acute rejection. Similarly, Kamar et al. (35) performed a RCT focusing on induction therapy. They found that both ATG and basiliximab were safe, with no significant difference in the overall recipient survival rates. In a RCT of pediatric liver transplantation, Dong et al. (36) found that basiliximab induction therapy is a safe and effective strategy that reduces acute rejection without compromising post-operative graft survival rates. Basiliximab selectively blocks T-cell activation without causing depletion. This intrinsic mechanism underlies its relatively mild anti-rejection effect and is responsible for its minimal risk of infection, which likely contributes to higher long-term patient survival rates (31). The pharmacological profile of basiliximab indicates that in low-immunogenicity organ transplants (e.g., liver transplantation, combined liver-kidney transplantation, combined heart-liver transplantation), basiliximab should be considered the induction therapy of choice for recipients with a high degree of HLA matching and/or those at potential risk of infection. Concurrently, clinicians must diligently monitor for the occurrence of early rejection episodes and promptly adjust immunosuppressive therapy if necessary.

### 4.5 Mechanisms of belimumab-mediated immunosuppression

Belimumab binds to and antagonizes the soluble BLyS protein, a cytokine pivotal for promoting B-cell survival and proliferation while contributing to the plasma cell niche (37). Its mechanism of action involves binding to tumor necrosis factor receptor superfamily, thereby effectively obstructing the interaction between BLyS and its receptor (38). By engaging the TNF receptor, belimumab inhibits the survival, maturation, and activation of B lymphocytes, thereby preventing their differentiation into plasma cells. Furthermore, it impedes both T lymphocyte-dependent and T lymphocyte-independent antibody responses, as well as T lymphocyte costimulation (39). A growing body of evidence suggests that regulatory B cells play a critical role in transplantation. First, the association between anti-CD20 therapy (rituximab) and T-cell mediated rejection has been documented (40). Second, renal transplantation recipients exhibiting an increased presence of migratory B cells demonstrated a decrease in rejection episodes (41). Lastly, experimental models indicate that inhibition of BLyS effectively prevents rejection and influences the survival of allografts (42). Conversely, some studies have shown that anti-BLyS therapy is ineffective in reducing DSA concentrations (43). Gemma D (44) et al. conducted a RCT comparing the efficacy and safety of belimumab to that of a placebo. The results revealed that belimumab significantly reduced the levels of immature B cells, activated memory B cells, circulating plasma cells, and DSA without increasing the risk of infection in recipients. Currently, belimumab is widely used to treat systemic lupus erythematosus (SLE). Mariette et al. (45) conducted a phase II RCT to compare the efficacy and safety of the combination of belimumab and rituximab monotherapy. Their conclusions indicated consistency in safety; however, the combination demonstrated a significantly greater effect on efficacy. Additionally, Zhang et al. (46) conducted a phase III open-label observational study, primarily assessing the safety of belimumab, with secondary endpoints focused on evaluating its efficacy. During a period of six years, belimumab was found to be both safe and effective for treating SLE. Despite the relative scarcity of research on belimumab in the context of SOT induction therapy, substantial evidence supports its safety and efficacy in SLE treatment. Future investigations should conduct prospective multicenter RCTs to validate belimumab in the context of organ transplantation induction therapy, as it may represent a promising strategy in this field.

### 5.6 Limitations

Although this study included 68 RCTs involving 9,626 patients, several limitations must be addressed. First, the number of RCTs examining certain intervention measures was limited and not all RCTs strictly follow a dual-phase adaptive design. Second, the sample sizes in several of these trials were relatively small, which may have introduced bias into our conclusions. Third, the pooled analysis of induction therapy for various solid organs may result in some degree of heterogeneity, necessitating subsequent validation in subgroup analyses. As emphasized in this article, the most effective approach to demonstrating the efficacy and safety of pharmacological agents is through well-designed, prospective multicenter RCTs with substantial sample sizes, which provide robust evidence necessary for clinical decision-making and regulatory approval. Therefore, these findings should be interpreted with caution.

## 5 Conclusion

Alemtuzumab emerged as the optimal therapy for minimizing rejection, while OKT3 and basiliximab were superior for graft and overall survival, respectively. Belimumab exhibited the strongest potential for reducing incidence of infection. These findings highlight therapy-specific advantages for optimizing SOT outcomes.

## Data Availability

The original contributions presented in the study are included in the article/**Supplementary Material**. Further inquiries can be directed to the corresponding author.
