# Supplementary material for: Comparative efficacy and safety of induction therapy in solid organ transplantation: a systematic review and network meta-analysis
Source: Front Immunol. 2025 Jul 14;16:1625710. doi: 10.3389/fimmu.2025.1625710 (PMC12301192; doi:10.3389/fimmu.2025.1625710)
Supplement: Supplementary file 1 [file DataSheet1.docx]

Supplementary Material 1. The specific search strategy.

PubMed search strategy

1. (((((((((((((Graft Rejection[MeSH Terms]) ) OR (Graft Rejection[Title/Abstract])) OR (Graft Rejections[Title/Abstract])) OR (Rejection, Graft[Title/Abstract])) OR (Rejections, Graft[Title/Abstract])) OR (Rejection, Transplant[Title/Abstract])) OR (Rejections, Transplant[Title/Abstract])) OR (Transplant Rejections[Title/Abstract])) OR (Transplant Rejection[Title/Abstract])) OR (Transplantation Rejection[Title/Abstract])) OR (Rejections, Transplantation[Title/Abstract])) OR (Rejection, Transplantation[Title/Abstract])) OR (Transplantation Rejections[Title/Abstract])randomized controlled trial[Publication Type] OR randomized[Title/Abstract] OR placebo[Title/Abstract]
2. ((Transplantation[MeSH Terms]) OR (Transplantation[Title/Abstract])) OR (Transplantations[Title/Abstract])
3. randomized controlled trial[Publication Type] OR randomized[Title/Abstract] OR placebo[Title/Abstract]
4. (((((((((((((((Graft Rejection[MeSH Terms]) ) OR (Graft Rejection[Title/Abstract])) OR (Graft Rejections[Title/Abstract])) OR (Rejection, Graft[Title/Abstract])) OR (Rejections, Graft[Title/Abstract])) OR (Rejection, Transplant[Title/Abstract])) OR (Rejections, Transplant[Title/Abstract])) OR (Transplant Rejections[Title/Abstract])) OR (Transplant Rejection[Title/Abstract])) OR (Transplantation Rejection[Title/Abstract])) OR (Rejections, Transplantation[Title/Abstract])) OR (Rejection, Transplantation[Title/Abstract])) OR (Transplantation Rejections[Title/Abstract])) AND (((Transplantation[MeSH Terms]) OR (Transplantation[Title/Abstract])) OR (Transplantations[Title/Abstract]))) AND (randomized controlled trial[Publication Type] OR randomized[Title/Abstract] OR placebo[Title/Abstract])

Embases search strategy

1. 'graft rejection'/exp
2. 'graft rejection':ab,ti
3. 'graft rejections':ab,ti
4. 'rejection, graft':ab,ti
5. 'rejections, graft':ab,ti
6. 'rejection, transplant':ab,ti
7. 'rejections, transplant':ab,ti
8. 'transplant rejections':ab,ti
9. 'transplant rejection':ab,ti
10. 'transplantation rejection':ab,ti
11. 'rejections, transplantation':ab,ti
12. 'rejection, transplantation':ab,ti
13. 'transplantation rejections':ab,ti
14. 'transplantation rejections':ab,ti
15. 'transplantation rejections':ab,ti
16. #1 OR #2 OR #3 OR #4 OR #5 OR #6 OR #7 OR #8 OR #9 OR #10 OR #11 OR #12 OR #13 OR #14 OR #15
17. 'transplantation'/exp
18. 'transplantation':ab,ti
19. 'transplantations':ab,ti
20. #17 OR #18 OR #19
21. 'double-blind':ab,ti
22. 'placebo':ab,ti
23. 'random':ab,ti
24. #21 OR #22 OR #23
25. #16 AND #20 AND #24

WOB search strategy

1. TS=(Graft Rejection) and Preprint Citation Index (Exclude – Database)
2. TS=(Graft Rejections) and Preprint Citation Index (Exclude – Database)
3. TS=(Rejection, Graft) and Preprint Citation Index (Exclude – Database)
4. TS=(Rejections, Graft) and Preprint Citation Index (Exclude – Database)
5. TS=(Rejection, Transplant) and Preprint Citation Index (Exclude – Database)
6. TS=(Rejections, Transplant) and Preprint Citation Index (Exclude – Database)
7. TS=(Transplant Rejections) and Preprint Citation Index (Exclude – Database)
8. TS=(Transplant Rejection) and Preprint Citation Index (Exclude – Database)
9. TS=(Transplantation Rejection) and Preprint Citation Index (Exclude – Databas
10. TS=(Rejections, Transplantation) and Preprint Citation Index (Exclude – Database
11. TS=(Rejection, Transplantation) and Preprint Citation Index (Exclude – Database)
12. TS=(Transplantation Rejections) and Preprint Citation Index (Exclude – Database)
13. #12 OR #11 OR #10 OR #9 OR #8 OR #7 OR #6 OR #5 OR #4 OR #3 OR #2 OR #1 and Preprint Citation Index (Exclude – Database)
14. TS=(Transplantation) and Preprint Citation Index (Exclude – Database)
15. TS=(Transplantations) and Preprint Citation Index (Exclude – Database)
16. #15 OR #14 and Preprint Citation Index (Exclude – Database)
17. TS=(random) and Preprint Citation Index (Exclude – Database)
18. TS=(placebo) and Preprint Citation Index (Exclude – Database)
19. TS=(double-blind) and Preprint Citation Index (Exclude – Database)
20. TS=(randomized controlled trial) and Preprint Citation Index (Exclude – Database)
21. TS=(randomized) and Preprint Citation Index (Exclude – Database)
22. TS=(placebo) and Preprint Citation Index (Exclude – Database)
23. #22 OR #21 OR #20 OR #19 OR #18 OR #17 and Preprint Citation Index (Exclude – Database)
24. #23 AND #16 AND #13 and Preprint Citation Index (Exclude – Database)

Cochrane Library search strategy

1. (Graft Rejection):ti,ab,kw OR (Graft Rejections):ti,ab,kw OR (Transplant Rejections):ti,ab,kw OR (Rejections, Transplantation):ti,ab,kw OR (Rejections, Transplantation):ti,ab,kw
2. (Transplantation):ti,ab,kw OR (Transplantations):ti,ab,kw
3. #1 AND #2

Supplementary Material 2. The Deviance Information Criterion.

|  | consistency | non-consistency |
| --- | --- | --- |
| Rejection rate | 246.41801 | 251.35501 |
| Graft survival rate | 168.36069 | 173.19347 |
| Overall survival rate | 207.03146 | 212.00225 |
| Infection rate | 152.98069 | 155.22854 |
